# CDC Safety Training Course for Ebola Virus Disease Healthcare Workers

**DOI:** 10.3201/eid2313.170549

**Published:** 2017-12

**Authors:** Rupa Narra, Jeremy Sobel, Catherine Piper, Deborah Gould, Nahid Bhadelia, Mary Dott, Anthony Fiore, William A. Fischer, Mary Jo Frawley, Patricia M. Griffin, Douglas Hamilton, Barbara Mahon, Satish K. Pillai, Emily F. Veltus, Robert Tauxe, Michael Jhung

**Affiliations:** Centers for Disease Control and Prevention, Atlanta, Georgia, USA (R. Narra, J. Sobel, C. Piper, D. Gould, M. Dott, A. Fiore, P.M. Griffin, D. Hamilton, B. Mahon, S.K. Pillai, R. Tauxe, M. Jhung);; Boston University School of Medicine, Boston, Massachusetts, USA (N. Bhadelia);; The University of North Carolina at Chapel Hill, Chapel Hill, North Carolina, USA (W.A. Fischer II);; Médecins Sans Frontières, New York, New York, USA (M.J. Frawley, E.F. Veltus)

**Keywords:** Ebola, epidemic, training, course, infection control, emergency response, viruses, United States, Africa, global health security, EVD, Ebola virus disease

## Abstract

Response to sudden epidemic infectious disease emergencies can demand intensive and specialized training, as demonstrated in 2014 when Ebola virus disease (EVD) rapidly spread throughout West Africa. The medical community quickly became overwhelmed because of limited staff, supplies, and Ebola treatment units (ETUs). Because a mechanism to rapidly increase trained healthcare workers was needed, the US Centers for Disease Control and Prevention developed and implemented an introductory EVD safety training course to prepare US healthcare workers to work in West Africa ETUs. The goal was to teach principles and practices of safely providing patient care and was delivered through lectures, small-group breakout sessions, and practical exercises. During September 2014–March 2015, a total of 570 participants were trained during 16 course sessions. This course quickly increased the number of clinicians who could provide care in West Africa ETUs, showing the feasibility of rapidly developing and implementing training in response to a public health emergency.

In 2014, epidemic Ebola virus disease (EVD) rapidly spread throughout West Africa; by August of that year, ≈2,600 EVD cases and 1,400 deaths had been reported (*1*). Widespread EVD transmission occurred in Guinea, Sierra Leone, and Liberia for several reasons. First, these countries had undergone years of civil war and unrest, which damaged an already fragile healthcare infrastructure and reduced the healthcare workforce (*2**–**4*), gravely limiting the countries’ ability to rapidly respond to a growing epidemic (*5*). Second, EVD is a hemorrhagic fever readily transmissible in the absence of rigorous infection prevention and control (IPC) (*6*). Ebola virus is spread by direct contact with body fluids of patients or contaminated fomites (*7*). For outbreak control, isolation of patients from the community is essential (*8*). EVD patients can arrive at healthcare facilities with severe symptoms such as substantial dehydration from vomiting, diarrhea, or hemorrhage, requiring aggressive intravenous resuscitation (*9*). Third, the EVD epidemic placed medical workers themselves at risk. Few healthcare workers have cared for patients with such a severe and highly transmissible disease requiring this degree of stringent IPC. The close patient interactions that were needed put healthcare workers at risk for infection (*9*,*10*). 

Personal protective equipment (PPE) serves as a physical barrier and can protect healthcare workers when used properly. However, PPE is only one IPC measure used to protect healthcare workers from EVD (*11*). Moreover, availability of PPE alone is not adequate for preventing infection. Without strict adherence to the complex processes of donning and doffing PPE and proper conduct while wearing PPE, transmission can still occur. Improper donning and doffing of PPE can result in self-contamination if unprotected mucous membranes or broken skin are exposed to infected body fluids (*12*). PPE doffing, in particular, carries high risk for self-contamination because of its complexity combined with healthcare worker fatigue after tiring shifts in an ETU (*12**–**14*). Fourth, the setting of this epidemic was unusual. Unlike previous Ebola outbreaks, which occurred predominantly in rural areas, the 2014 EVD epidemic occurred primarily in densely populated urban areas. Previous rural outbreaks had been controlled by isolating EVD patients from the community through early admission to healthcare facilities capable of managing the disease. In 2014, the rapid increase in the number of EVD patients early in the epidemic quickly overwhelmed the number of trained clinicians and healthcare facilities that could care for them (*5*).

By late August 2014, a total of 240 registered healthcare workers had acquired EVD and 120 had died (*15*). As the number of infected healthcare workers rose, medical staff became increasingly fearful of contracting EVD from patients. The World Health Organization (WHO) found risk of contracting EVD during this epidemic to be 21–32 times higher among healthcare workers than among non–healthcare workers (*16*). Some clinics and hospitals closed because of staff shortages or healthcare workers’ unwillingness to work, exacerbating the lack of facilities (*17*).

Transmission models developed by the US Centers for Disease Control and Prevention (CDC) indicated that to halt the epidemic, ≈70% of EVD patients should be isolated in appropriate treatment facilities (*18*). The models projected that if transmission were not rapidly reduced, EVD cases in Liberia and Sierra Leone could reach 550,000 by January 2015 (*18*). A key component of the international response to the epidemic entailed deploying trained volunteer healthcare workers to EVD-affected areas to reduce community transmission by isolating EVD patients and providing care in a safe healthcare setting. To support this urgent need, CDC developed and implemented an introductory EVD safety training course to prepare volunteer US healthcare workers to work in West Africa Ebola treatment units (ETUs).

Few deploying clinicians had been trained in the infection control practices needed to provide EVD care safely in limited-resource settings, which are distinctly different from US hospitals. In August 2014, the only structured EVD training for healthcare workers was a 2-day course held in Brussels, Belgium, by Médecins Sans Frontières (MSF) (*19*). MSF acquired extensive EVD care experience in Africa and developed this course to share knowledge with staff deploying to respond to the epidemic (*20**–**22*). Given the urgency and need for international healthcare volunteers, demand quickly exceeded course availability. On August 26–27, 2014, three CDC members attended the MSF course in Brussels in anticipation of developing a US-based version of the training. After rapid course planning and development, CDC launched its first EVD Safety Training Course in Anniston, Alabama, USA, on September 22, 2014. We summarize the development and operation of the course.

## Course Concept

The course objective was to introduce deploying healthcare workers to principles and practices of safely providing patient care in a West Africa ETU. Key learning objectives included understanding of the following: EVD modes of transmission, ETU structure and operation, ETU IPC procedures (proper PPE donning and doffing techniques, disinfection, sharps and waste management), and personal safety within ETUs (psychologic preparation, stress management, overheating while wearing PPE). The various organizations with which trainees would deploy stocked different types of PPE. Thus, our training strategy centered on teaching sound principles and methods to prevent disease transmission, rather than focusing on a particular type of PPE or protocol. We wanted to prepare volunteers for the complex and changing clinical and social environment in the center of a transmissible disease epidemic of unprecedented scope and severity. The course included classroom instruction and practical hands-on training in a realistically constructed mock ETU. At the time, West Africa ETUs were simple healthcare isolation units that combined a specific layout with rigorous IPC practices and offered patient isolation, diagnosis, and oral and intravenous rehydration therapy and medications. Therefore, we focused clinical management instruction on these topics. 

The course provided introductory training as the first stage of a more comprehensive process, which involved further in-country mentoring under direct supervision of local or international staff with previous EVD experience. We designed a sustainable, repeatable course model that enabled efficient course implementation by sequential cohorts of instructors. Beginning in September 2014, the US-based 3-day course was offered weekly at the same location.

## Staff and Setting

The initial course design team was a multidisciplinary 15-person unit comprising members who had attended the MSF Brussels course, infectious disease physicians, medical epidemiologists, instructional designers, and healthcare workers recently deployed to West Africa who had worked in ETUs or EVD-affected communities (returning responders). Course development incorporated input from experts in public health and EVD from CDC, MSF, and WHO and from US-based infection control experts. When the pilot course was launched, the team had grown to a 40-person unit including data managers, communication specialists, and logisticians.

The course was held at the US Federal Emergency Management Agency (FEMA) Center for Domestic Preparedness (CDP) in Anniston (https://cdp.dhs.gov/). CDP is an all-hazards training center equipped with classrooms, audiovisual equipment, dormitory-style lodging, and food and transportation services. The 124-acre campus has buildings and outdoor spaces well suited for the construction of austere mock West Africa ETUs for simulated exercises. CDP trains ≈45,000 emergency responders yearly and efficiently supported the rapidly expanding course. The location, 90 miles from CDC headquarters in Atlanta, Georgia, USA, enabled relatively convenient transportation of staff, supplies, and trainees.

## Trainees

We assigned high priority to US healthcare workers scheduled to deploy to West Africa. We required that trainees have a license to provide clinical care, recent experience providing direct patient care, and affiliation with a governmental or nongovernmental organization responsible for travel to and from West Africa. Healthcare workers included nurses, physicians, paramedics, physician assistants, and others who would work directly with EVD patients in ETUs (Table 1). Additional participants included representatives of organizations who were interested in designing similar courses or assessing the course’s suitability for their deploying staff.

**Table 1:** Professions of 570 trainees attending Ebola Virus Disease Safety Training Course, Anniston, Alabama, USA, 2014–2015

Profession	No. (%)
Healthcare worker	387 (68)
Nurse	180 (32)
Physician	169 (30)
Physician assistant/nurse practitioner	20 (3)
Paramedic/emergency medical technician	18 (3)
Non–clinical care provider	185 (32)
Public health official	44 (8)
Pharmacist	25 (4)
Scientist	21 (4)
Mental health professional	17 (3)
Other	76 (13)

### Operations and Logistics

**Figure 1:**
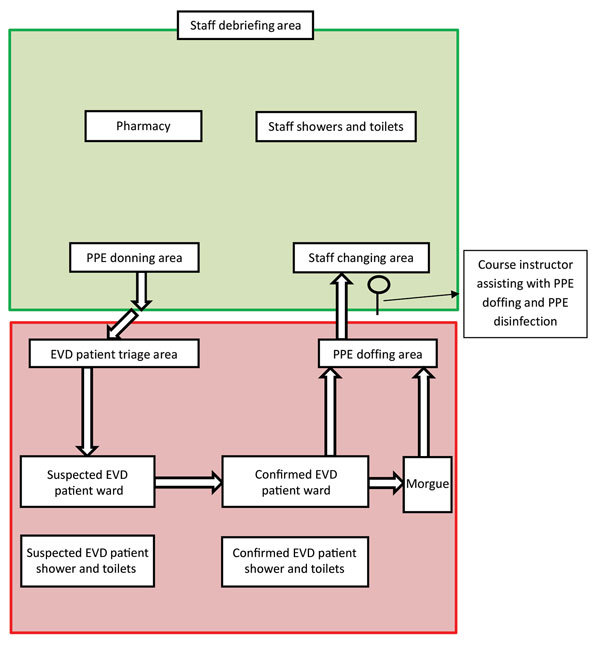
Layout of mock Ebola Treatment Unit used during the Centers for Disease Control and Prevention Ebola Safety Training Course, held at the US Federal Emergency Management Agency Center for Domestic Preparedness in Anniston, Alabama, USA, 2014–2015. Green indicates low-risk zone, which included staff PPE donning area, the staff changing area (after PPE doffing), pharmacy, staff showers and toilets, and a staff debriefing area; red indicates high-risk zone, which included EVD patient triage area, wards for patients with suspected and confirmed EVD, patient showers and toilets, and the morgue. Arrows indicate staff unidirectional movement from lower to higher risk zones. EVD, Ebola virus disease; PPE, personal protective equipment.

The 3-day course consisted of lectures, small-group discussions, and practical exercises requiring trainees to perform simulated patient care activities in a mock ETU (Figure 1). Course days lasted ≈9 hours. During September 2014**–**March 2015, a total of 16 courses were held. Trainees traveled to Atlanta independently; CDC provided bus transport from Atlanta to Anniston, private dormitory rooms, on-site transport, and 3 meals per day. The environment promoted easy monitoring of trainees and emotional bonding and support among course participants.

The most valuable supplies for the course, and the most challenging to obtain, were PPE. Other materials were supplied by CDP or purchased locally. A list of supplies can be found at http://www.cdc.gov/vhf/ebola/hcp/safety-training-course/training-toolkit.html.

**Figure 2:**
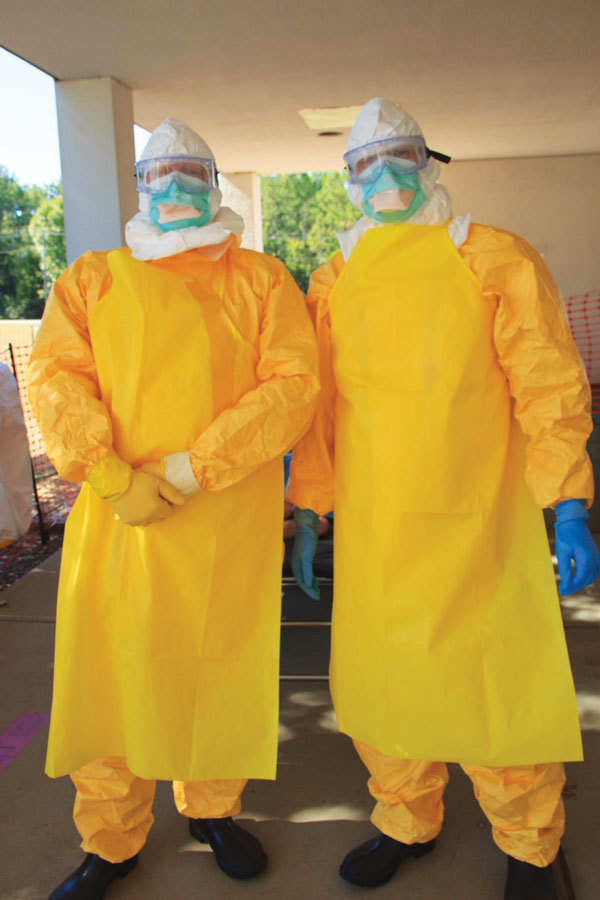
Example of personal protective equipment (PPE) used during the Centers for Disease Control and Prevention Ebola Safety Training Course, held at the US Federal Emergency Management Agency Center for Domestic Preparedness in Anniston, Alabama, USA, 2014–2015. From top to bottom: head covering, eye protection, N95 respirator, apron over coverall, 2 pairs of latex gloves, gum boots.

PPE for trainees consisted of coverall (protective suit), eye protection (goggles or full face shield), N95 respirator or surgical mask, 2 pairs of latex gloves, hood covering the head and neck, apron, gum boots, and surgical scrubs (Figure 2). PPE procurement was challenging for 2 reasons. First, protocols dictating which PPE supplies were needed had to be established. However, in 2014, consensus on optimal PPE for use in West Africa ETUs was lacking (*23*). Consequently, experts from CDC, MSF, and WHO used preexisting MSF and WHO PPE guidelines to develop protocols for the course (*24*,*25*). Protocols balanced the anticipated availability of specific PPE in West Africa with safe IPC practices. The goal was to impart a fundamental understanding of infection control measures necessary to avoid self-contamination and assess the safety of PPE that trainees might encounter in West Africa ETUs. Within 2 weeks, we procured a combination of MSF- and WHO-style PPE and supplies from local manufacturers, international distributors, and medical supply companies. Second, worldwide shortages of fluid-resistant coveralls and specially made hoods required rapid substitutions to best emulate what participants might encounter in West Africa ETUs (*26*). To conserve PPE in short supply, over the 3-day course, trainees reused fluid-resistant suits and aprons.

## Course Content

As the course development team, we drew course content from materials from MSF, WHO, and CDC. We referenced technical manuals (*27*,*28*), online resources, videos, and other materials from the MSF Brussels EVD course, as well as input from returning responders and Ebola experts. Course materials included lectures, EVD case scenarios, step-by-step PPE protocols, and practical exercise instruction. Course materials underwent CDC institutional clearance, which entailed detailed review of each topic by CDC-designated experts, and were made available to trainees in paper and electronic formats.

Because healthcare workers in West Africa needed to strictly adhere to infection control principles to minimize the risk of contracting EVD, we focused most course content on IPC. Crucial IPC components for preventing EVD transmission are methodical PPE donning and doffing, proper patient flow and triage, injection and sharps safety, environmental cleaning and waste disposal, safe handling of laboratory samples, and safe management of the dead (*11*). We taught these principles through lectures, small-group breakout sessions, and practical exercises.

### Lectures and Classroom Exercises

Morning sessions were devoted to lectures and small-group activities. Lecture topics included EVD epidemiology, transmission, and pathophysiology; elementary clinical management of patients; IPC; proper ETU design; disinfection and waste management in ETUs; mental health resilience; occupational health; community health promotion; and experimental treatments and vaccines for EVD. Small-group activities consisted of discussions with recently returned EVD responders and a series of tabletop exercises: 1) interactive case studies on EVD recognition and triage; 2) designing safe ETUs, including patient care areas, placement of handwashing stations, and healthcare worker flow; and 3) cultural sensitivity exercises, including techniques for interacting with community members while recognizing and respecting local customs.

### Exercises in a Mock ETU

Afternoon sessions consisted of practical exercises that involved real-life scenarios, which comprised 50% of the course. Practical exercises requiring trainees to be in full PPE were a foundation of this course. We focused on repetitive practical exercises involving donning PPE with a partner, performing simulated high-risk patient-care activities, and doffing PPE under close supervision. Anecdotal observations indicated that trainees entering the mock ETU experienced increased concentration and anxiety, suggesting a level of realism in the simulated training setting.

**Figure 3:**
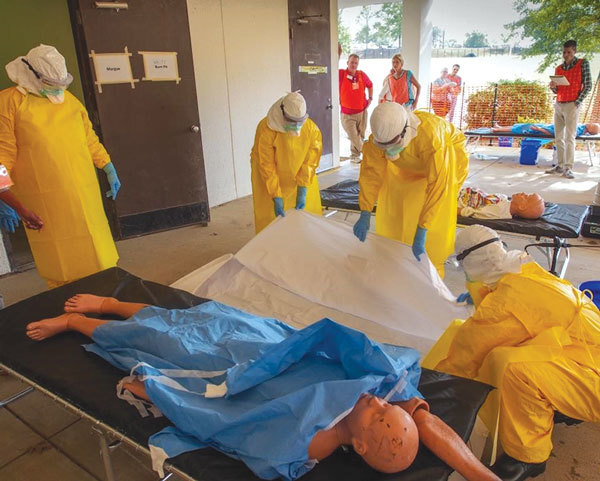
Constructed mock Ebola Treatment Unit used during the Centers for Disease Control and Prevention Ebola Safety Training Course, held at the US Federal Emergency Management Agency Center for Domestic Preparedness in Anniston, Alabama, USA, 2014–2015. Trainees prepare to place a simulated deceased patient into a body bag.

In West Africa, healthcare workers faced additional challenges of harsh conditions, such as high temperatures, inconsistent electricity, poor lighting and visibility, and overcrowded ETUs (*12*). Returning responders described overworked staff in West Africa, covered in layers of PPE in sweltering heat, who experienced excessive sweating, dehydration, fogged eye protection, and decreased dexterity while caring for and transporting critically ill and dying patients. As core body temperatures rise while wearing PPE, overheating can lead to motor and cognitive impairment, further increasing healthcare worker vulnerability to breaches of safety practices (*29*). Thus, we constructed 2 mock ETUs to simulate the challenging conditions trainees might face in West Africa. Our mock ETUs had clearly designated low- and high-risk zones, stocks of PPE with changing areas, simulated chlorine footbaths and handwashing stations, weighted patient dummies, a triage area, and a unidirectional flow pattern from low- to high-risk zones (Figures 1,3).

Teams of 4–6 trainees entered mock ETUs, where they received a focused orientation and then donned PPE under direct supervision of a course instructor. According to MSF protocol, we taught a buddy system during practical exercises, whereby partners observed each other during PPE donning and regularly checked for breaches in PPE or infection control protocol. Trainees then entered the patient-care area, where they conducted instructor-guided simulated patient-care activities, including collecting and preparing blood specimens for transport, transporting a patient into the ETU, performing environmental decontamination and waste management, and transporting a deceased patient from a patient care area to a morgue. After these activities, trainees learned a regimented doffing process in a designated area of the mock ETU, performing the structured PPE removal sequence under direct supervision of course instructors and the observing partner.

## Course Evaluation

### Trainee Demographics

By March 25, 2015, 570 trainees attended a total of 16 course sessions. Trainees came from US governmental agencies (n = 352, 62%); 43 nongovernmental organizations, (n = 164, 29%); and other organizations, including foreign governments, private healthcare organizations, and academic institutions (n = 54, 9%) (Table 2). Trainees traveled from 36 states and 20 countries to attend the course. To our knowledge, although most deployed to ETUs in West Africa, some for months at a time, none of the trainees acquired EVD during deployment.

**Table 2:** Sponsoring agencies of 570 trainees attending Ebola Virus Disease Safety Training Course, Anniston, Alabama, USA, 2014–2015

Agency	No. (%)
US government	352 (62)
Public Health Service	296 (52)
Centers for Disease Control and Prevention	26 (5)
Armed Forces	18 (3)
Other	12 (2)
Nongovernmental organizations	164 (29)
Partners in Health	38 (7)
Samaritan’s Purse	24 (4)
International Medical Corps	15 (3)
Americares	11 (2)
Other	76 (13)
Academic institutions, foreign governments, and other	54 (9)
	

### Costs and Staff Resources

A course of this scale required substantial resources. As the course evolved, the number of trainees increased each week. To ensure close supervision during practical exercises, we added course graduates to the staff to maintain an instructor:trainee ratio of 1:4. Over the life of the course, a total of 193 staff (89 CDC, 104 non-CDC) provided the training: 26 experts in infectious diseases and 117 practical exercise course instructors.

We estimate that 30,000 staff person-hours were required for course development (12,000 hours), 16 sessions of course instruction (10,000 hours), and course material revision (8,000 hours). The average total cost for a 3-day course was approximately US$27,000, or $750 per trainee for meals, lodging, transport, administrative coordination, and PPE and other supplies (Table 3). Course development and implementation relied on a multidisciplinary team; outside experts with ETU experience were essential. Because no mechanism existed to rapidly establish a training course of this scope, assembling and maintaining this large, diverse team was time-consuming and challenging. Institutional support was critical for creating interagency collaborations. Modifying an existing interagency agreement with the Oak Ridge Institute for Scientific Education (https://orise.orau.gov/) was instrumental in finding and supporting the travel of many trainers, and a new interagency agreement between CDC and FEMA provided access to the CDP campus and infrastructure.

**Table 3:** Estimated cost per Ebola Virus Disease Safety Training Course, Anniston, Alabama, USA, 2014–2015*

Expense	Cost, US$
Meals	5,182.92
Lodging	5,700.00
Administrative and program costs	4,386.24
Transportation	5,355.00

Personal protective equipment	6,468.32
Total	27,092.48

*3-day course, 36 trainees.

## Feedback and Observations

Feedback from course graduates and returning responders during and immediately after each course session confirmed that the most crucial aspects of the course were hands-on, practical exercises, especially donning and doffing PPE. We therefore constructed a second mock ETU where students were able to don and doff PPE, practice dexterity exercises in double-gloved hands, and develop and discuss other potential ETU layouts while waiting to perform the practical exercises in the main ETU. This second mock ETU enabled trainees to practice, ask additional questions, and further discuss infection control procedures.

To improve trainees’ understanding of the systematic process, our teaching model also incorporated trainees as instructors during the practical exercises. We asked trainees to identify breaches in their partner’s PPE and instruct fellow trainees during the doffing process. To better understand PPE doffing, trainees replaced course instructors during the doffing process and gave explicit step-by-step instructions to fellow trainees as they removed PPE piece by piece. To ensure that proper techniques and procedures were followed, course instructors supervised all activities.

We encouraged flexibility in course instructors and trainees in various scenarios but still stressed the value of recognizing a safe work environment. International support for control of the 2014 West Africa EVD epidemic entailed aid from hundreds of international organizations. Healthcare workers who deployed to West Africa therefore encountered a wide variety of PPE supplies, ETU layouts, and safety protocols. Hence, rather than focusing our training on mastering a specific protocol, we attempted to instill a general culture of safety by providing trainees with the knowledge and skills to work safely in ETUs, identify and correct safety deficiencies, and feel empowered to withdraw from unsafe situations.

## Course Sustainability

Given the relative rarity of EVD, limited formal training courses exist worldwide. Several organizations and institutions, including foreign ministries of health, have expressed interest in establishing their own EVD training courses and requested our training materials. In response to these requests and to make course content easily accessible and reproducible, we created a Web-based toolkit that included all lectures, facilitator guides for small-group exercises, comprehensive trainer guides with video tutorials of practical exercises, supply checklists, and administrative templates required to implement the course. The toolkit went through extensive review and clearance by representatives of CDC, MSF, and WHO; on April 2, 2015, the complete toolkit was posted on the CDC Ebola website (https://www.cdc.gov/vhf/ebola/hcp/safety-training-course/training-toolkit.html). The step-by-step instructions and detailed materials in the toolkit might enable other organizations and countries to reproduce this training, given appropriate resources. The kit could help other countries, particularly those with a history of EVD outbreaks, better prepare for and respond to future outbreaks.

## Conclusions

Establishment of the CDC EVD Safety Training Course was a relatively low-cost but high-impact activity that required an exceptional time commitment and flexibility from an evolving multidisciplinary team and dedicated trainees. Effective course execution required staff with diverse specialties, specialized supplies, transportation and housing for trainees, specific facilities for training, rapid access to funding, and complex interagency agreements. The implementation challenges included rapid hiring, contracting, and management of nearly 200 staff; recruitment and selection of course trainees and instructors; and development and review of course materials, including PPE protocols for ETUs in West Africa, when no international consensus existed.

Sudden public health emergencies can demand intensive and specialized training. The CDC EVD Safety Training Course was an innovative and extensive US training effort designed specifically to fill the previously unmet need to prepare clinicians to deploy to West Africa in response to the 2014 EVD epidemic. As was the case for the Haiti cholera epidemic of 2010, rapid development of a specialized clinical training course was a fundamental component of the public health response to epidemic disease (*30*). The CDC EVD Safety Training Course quickly increased the number of clinicians who could provide care in West Africa ETUs, showing the feasibility of rapidly developing and implementing focused training in response to a public health emergency. Course graduates could use these specialized skills for future outbreaks of hemorrhagic fevers, although setting-specific components of the course addressing epidemiologic, cultural, and other issues would need to be adapted. Moreover, several key components of the EVD Safety Training Course (i.e., IPC procedures such as proper PPE donning and doffing, personal safety measures such as stress management in ETUs) might have considerable applicability to outbreaks of other pathogens that affect resource-limited settings.

In the following ways, advance preparation could greatly help rapidly mobilize a multifaceted training course as part of the response to future complex epidemic infectious disease emergencies. First, having a standing cadre of dedicated staff and a plan for developing training courses would increase the efficiency and speed of course development and implementation. The plan would include maintaining lists of staff specialized in instructional design, infectious diseases, public health, healthcare infection control, and logistics, who could fill needs depending on the course and contact lists of supplemental staff and contractors. Second, having contact and ordering information for various local and international manufacturers with detailed resource estimates could expedite supply procurement. Third, having established training sites with active partnership agreements in place would bypass the time-consuming and burdensome process of site identification and contract negotiation. Fourth, reliable funding sources and high-level institutional support is critical for quickly overcoming barriers.

The 2014 Ebola epidemic provides a reminder that the threat of global outbreaks of emerging infectious diseases is real and immediate. It is with these threats in mind that CDC and public health partners developed the Global Health Security Agenda (https://www.cdc.gov/globalhealth/security/index.htm), which supports capacity-building in ≈40 countries to prevent, detect, and respond to infectious disease threats. The CDC EVD Safety Training Course is a relevant and timely example of how international partners can work collaboratively to meet the Global Health Security Agenda objectives of more rapidly detecting, responding to, and controlling public health emergencies at their source and thereby enhancing global health security. Maintaining institutional memory of this effort, by establishing a core team of educators who could serve as a dedicated rapid training team, would help preserve expertise gained by development of this course, which in turn would enable a more nimble response to future urgent training needs with regard to new or emerging pathogens.
